# Artificial Intelligence in Lung Cancer Pathology Image Analysis

**DOI:** 10.3390/cancers11111673

**Published:** 2019-10-28

**Authors:** Shidan Wang, Donghan M. Yang, Ruichen Rong, Xiaowei Zhan, Junya Fujimoto, Hongyu Liu, John Minna, Ignacio Ivan Wistuba, Yang Xie, Guanghua Xiao

**Affiliations:** 1 Quantitative Biomedical Research Center, Department of Population and Data Sciences, University of Texas Southwestern Medical Center, Dallas, TX 75390, USA; Shidan.Wang@utsw.edu (S.W.); donghan.yang@utsw.edu (D.M.Y.); ruichen.rong@utsw.edu (R.R.); xiaowei.zhan@utsw.edu (X.Z.); Hongyu.Liu@utsw.edu (H.L.); Yang.xie@utsw.edu (Y.X.); 2 Department of Translational Molecular Pathology, University of Texas MD Anderson Cancer Center, Houston, TX 77030, USA; JFujimot@mdanderson.org (J.F.); iiwistuba@mdanderson.org (I.I.W.); 3 Harold C. Simmons Comprehensive Cancer Center, University of Texas Southwestern Medical Center, Dallas, TX 75390, USA; john.minna@utsw.edu; 4 Hamon Center for Therapeutic Oncology Research, UT Southwestern Medical Center, Dallas, TX 75390, USA; 5 Departments of Internal Medicine and Pharmacology, University of Texas Southwestern Medical Center, Dallas, TX 75390, USA; 6 Department of Bioinformatics, University of Texas Southwestern Medical Center, Dallas, TX 75390, USA

**Keywords:** lung cancer, deep learning, pathology image, computer-aided diagnosis, digital pathology, whole-slide imaging

## Abstract

Objective: Accurate diagnosis and prognosis are essential in lung cancer treatment selection and planning. With the rapid advance of medical imaging technology, whole slide imaging (WSI) in pathology is becoming a routine clinical procedure. An interplay of needs and challenges exists for computer-aided diagnosis based on accurate and efficient analysis of pathology images. Recently, artificial intelligence, especially deep learning, has shown great potential in pathology image analysis tasks such as tumor region identification, prognosis prediction, tumor microenvironment characterization, and metastasis detection. Materials and Methods: In this review, we aim to provide an overview of current and potential applications for AI methods in pathology image analysis, with an emphasis on lung cancer. Results: We outlined the current challenges and opportunities in lung cancer pathology image analysis, discussed the recent deep learning developments that could potentially impact digital pathology in lung cancer, and summarized the existing applications of deep learning algorithms in lung cancer diagnosis and prognosis. Discussion and Conclusion: With the advance of technology, digital pathology could have great potential impacts in lung cancer patient care. We point out some promising future directions for lung cancer pathology image analysis, including multi-task learning, transfer learning, and model interpretation.

## 1. Introduction

Lung cancer is the leading cause of death from cancer in the United States and around the world [1,2,3]. Disease progression and response to treatment in lung cancer vary widely among patients. Therefore, accurate diagnosis is crucial in treatment selection and planning for each lung cancer patient. The microscopic examination of tissue slides remains an essential step in cancer diagnosis. It requires the pathologist to recognize subtle histopathological patterns in the highly complex tissue images. This process is time-consuming, subjective, and generates considerable inter- and intra-observer variation [4,5]. Hematoxylin and eosin (H&E) staining is the most popular tissue staining method [6]; with the advance of technology, H&E-stained whole slide imaging (WSI) of tissue slides is becoming a routine clinical procedure and has generated a massive number of pathology images that capture histological details in high resolution. Currently, the limited capacity of pathology image analysis algorithms is creating a bottleneck in digital pathology, constraining the ability of this technology to have greater clinical impact.

Recent developments in pathology image analysis [7,8] have led to new algorithms and software tools for clinical diagnosis and research into disease mechanisms. In 2011, the seminal works by Beck et al. [9] and Yuan et al. [10] applied pathology image analysis to breast cancer prognosis. Since then, computer algorithms for pathology image analysis have been developed to facilitate cancer diagnosis [11,12,13,14,15], grading [16,17,18,19,20], and prognosis [21,22]. In 2017, two studies [21,22] demonstrated the feasibility of utilizing morphological features in pathology slides for lung cancer prognosis, which opened up an exciting new direction for refining lung cancer prognosis using computer-aided image analysis.

Recently, artificial intelligence (AI) has been demonstrating remarkable success in medical image analysis owing to the rapid progress of “deep learning” algorithms [23,24,25], which have shown increasing power to solve complex, real-world problems in computer vison and image analysis [23,24,25]. The development of advanced deep learning algorithms could empower pathology image analysis and assist pathologists in challenging diagnostic tasks such as identifying neoplasia, detecting tumor metastasis, and quantifying mitoses and inflammation.

In this review, we aimed to provide an overview of current and potential applications for AI methods in lung cancer pathology image analysis. We first outlined the current challenges and opportunities in lung cancer pathology image analysis. Next, we discussed the recent deep learning developments that could potentially impact digital pathology in lung cancer. Finally, we summarized the existing applications of deep learning algorithms in lung cancer diagnosis and prognosis.

## 2. Current Challenges and Opportunities in Lung Cancer Pathology Image Analysis

### 2.1. Diagnosis: Tumor Detection and Classification

**Figure 1:**
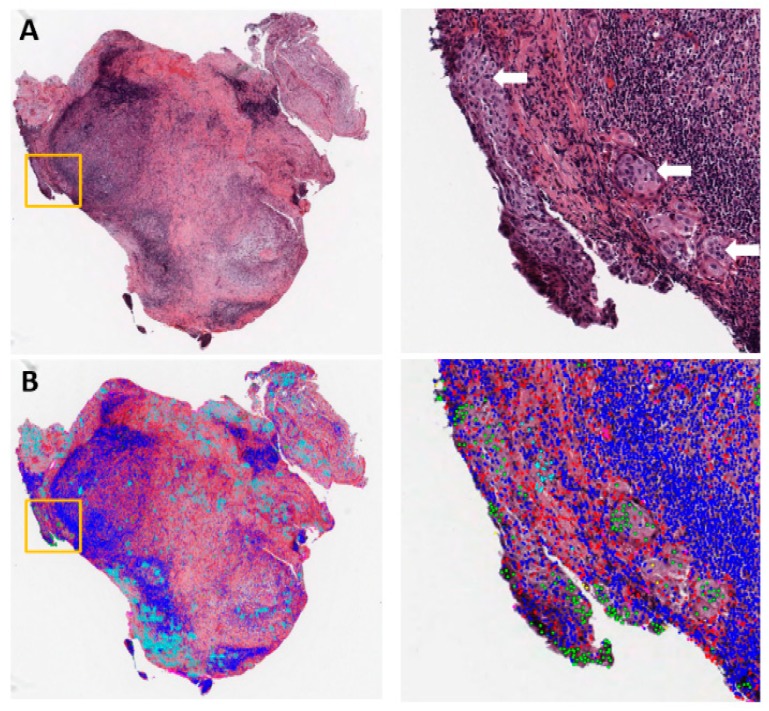
Example of metastasis detection in the lymph node for a lung adenocarcinoma patient. (**A**) Left: Hematoxylin and eosin (H&E)-stained lymph node pathology slide (40×). Data were collected by the National Lung Screening Trial (NLST). Tumor cells began to invade into the capsule in the orange box. Right: Region of interest in the orange box on the left, with white arrows pointing to tumor cells. (**B**) Cell classification result overlaid on the H&E image. Green: Tumor nuclei; blue: Lymphocytes; red: Stroma nuclei; cyan: Necrosis.

Pathology inspection of tissue slides is an important step in lung cancer diagnosis. For example, in the tumor, node, and metastasis (TNM) staging [26], the node (N) stage (regional lymph node involvement) is determined by examining whether the tumor has invaded the lymph nodes, based on pathology slides. Identifying the existence of tumor cells in dissected lymph nodes requires highly skilled pathologists and is a laborious task, especially if there are many dissected lymph nodes or the metastasis region(s) is small (Figure 1A). Computer-aided automatic detection of tumor cells in lymph nodes would greatly reduce the false negative rate, which would allow for better early detection and treatment of lung cancer, improve the accuracy of TNM staging, speed up the examination process, and reduce the workload for pathologists (Figure 1B).

Histological classification of tumor subtypes is another application of pathology image analysis in lung cancer diagnosis. According to each tumor’s histopathological features, lung cancer can be divided into non-small cell lung cancer (NSCLC) and small cell lung cancer (SCLC). NSCLC accounts for 85% of lung cancer cases and can be further separated into lung adenocarcinoma (ADC), squamous cell carcinoma (SCC) lung cancer, and large cell lung cancer. Each subtype of lung cancer has a distinct biological origin and mechanism(s). Lung ADC accounts for 50% of lung malignancies, with remarkable heterogeneity in clinical, radiological, molecular, and pathological features [27]. According to the growth pattern and morphology, ADC can be further divided into subgroups: Lepidic, papillary, acinar, micropapillary, and solid patterns [28]. These five morphology subtypes of lung ADC have different prognostic outcomes [29,30] and treatment responses [31]. Although extensive research has been conducted into ADC subtyping, classification of these subtypes remains a challenge for two main reasons: (1) poor consistency even among expert pathologists and (2) co-existence of multiple morphology subtypes across one or more slides from the same patient. As a result, for accurate diagnosis, the proportion of each subtype needs to be quantified, which can be a very challenging and time-consuming task for pathologists. Currently, common practice is to use the dominant subtype to represent the patient, which may result in a loss of information for other subtypes. Even to determine the dominant subtype of ADC, pathologists usually need to go through multiple tissue slides in order to provide an accurate diagnosis. Other tumor classification tasks for NSCLC, such as differential status classification [32] and ADC/SCC classification [22], are subject to similar technical challenges.

In addition, tumor spread through air spaces (STAS) has been demonstrated as a significant clinical factor associated with poor prognosis for tumor recurrence and patient survival [33]. Identification and quantification of STAS requires detailed inspection of whole tissue slides by experienced pathologists. Therefore, pathology image analysis tools for fast and accurate identification of STAS will be useful to pathologists.

### 2.2. Tumor Microenvironment (TME) Characterization Based on Substructure Segmentation

In addition to tumor grade and subtype [34], pathology images can provide insights into the TME. An essential step in the quantitative characterization of the TME is segmenting different types of tissue substructures and cells from pathology images. Such segmentation serves as the basis for various image analysis tasks, such as cell composition, spatial organization, and substructure-specific morphological properties. Lung cancer TME is mainly formed by tumor cells, lymphocytes, stromal cells, macrophages, blood vessels, etc. Studies in lung cancer have shown tumor-infiltrating lymphocytes (TIL) to be positive prognostic factors [35] and angiogenesis to be negatively associated with survival outcome [36], while stromal cells have complex prognostic effects [37,38]. Traditional image processing methods involve feature definition, feature extraction, and/or segmentation. Although these methods have been applied to segment lymphocytes and to analyze the spatial organization of TIL [39] and stromal cells [9] in the TME, quantitative characterization of lung cancer TME remains challenging for the following reasons: (1) The composition of lung cancer TME is complex and heterogeneous: In addition to the aforementioned cell types, other structures, including the bronchus, cartilage, and pleura, are often found in lung pathology slides. Such complexity and heterogeneity make segmentation and traditional feature definition a challenge. (2) Cell spatial organization (such as the spatial distributions of and interactions among different types of cells), despite their important roles in TME, are much more challenging to characterize than simply providing the numbers or proportions of different types of cells. Current studies mainly focus on the proportions of different types of cells, while ignoring the complex cell spatial organization, which could lead to limited and contradicting results about the roles of different types of cells in the TME. (3) For H&E-stained slides, the color may vary substantially depending on staining conditions and the time lapse between slide preparation and scanning. Traditional image processing methods based on handcrafted feature extraction cannot easily surmount these impediments. Robust methods adapted to diverse tissue structures and color conditions are required.

### 2.3. Prognosis and Precision Medicine

One of the most important potential applications of digital pathology in cancer is to predict patient prognosis and response to treatment based on histopathological or morphological phenotyping, in order to facilitate precision medicine. While specific pathological features, such as the aforementioned tumor grade and subtype, have been reported as significant prognostic factors, directly linking pathology images with survival outcomes remains largely unexplored. Similar opportunities exist in fully utilizing pathology images for predicting treatment response. In particular, immunotherapy has been shown to be effective for some NSCLC patients [40]. Although several genomic biomarkers, such as *PD-L1* expression [40] and neoantigen load [41], have been discovered to be predictive factors, immunotherapy response prediction remains a major challenge. The spatial organization of immune cells and the spatial distribution of TIL may be important factors in predicting immunotherapy response. However, automatic recognition of different types of cells and characterization of cell spatial organization are currently challenging tasks for pathology image analysis and require the development of new algorithms.

### 2.4. Association and Integration with Patient Genomic and Genetic Profiles

Another important emerging research field focuses on the relationship between patient genetic/genomic profiles and pathological/morphological phenotypes, which is essential for understanding the biological mechanisms underpinning cancer development and for selecting targeted therapy. Researchers have found an association between morphological features and tumor genetic mutations such as *EGFR* [42] and *KRAS* mutations [43]. The challenge in this area mainly lies in the facts that (1) both imaging and genomic/genetic data are extremely high-dimensional, and (2) the interaction between imaging and genomic features is largely unknown. Furthermore, molecular profiling data generate deep characterizations of the genetic, transcriptional, and proteomic events in tumors, while pathology images capture the tumor histology, growth pattern, and interactions with the surrounding microenvironment. Therefore, integrating imaging and molecular features could provide a more comprehensive view of individual tumors and potentially improve prediction of patient outcomes. However, how to construct and train a model that is capable of tackling such sophisticated data remains a difficult problem.

In summary, pathology image analysis is anticipated to become an important tool to facilitate precision medicine and basic research in cancer.

## 3. Advantages of Deep Learning Methods

To overcome the aforementioned challenges, various image processing and machine learning methods have been proposed and have so far achieved great progress. However, it is important to note the advantages of deep learning methods over non-deep-learning methods (also called shallow-learning methods).

**Figure 2:**
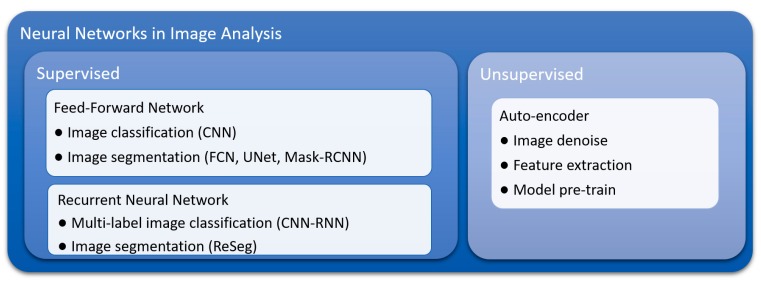
Taxonomy of common neural networks for image analysis. CNN: Convolutional neural network; FCN: Fully convolutional neural network; Mask-RCNN: Mask-regional convolutional neural network.

Currently, convolutional neural networks (CNNs) are the most frequently used deep learning model for image data classification, including tumor detection in pathology images of breast cancer [44,45], renal cell carcinoma [46], prostate cancer [47], and head and neck cancer [48]. Several forms of neural network have been derived from CNNs for image segmentation [49], including fully convolutional networks (FCNs) [50] and mask-regional convolutional neural networks (mask-RCNNs) [51]. Recurrent neural networks (RNNs), which are well known for modeling dynamic sequence behavior such as speech recognition, have also been explored in multi-label image classification [52] and image segmentation [53]. In additional to the aforementioned supervised deep learning models, autoencoder, an unsupervised deep learning model, has shown ability in analyzing pathology images through pre-training models [39], cell detection [54], and image feature extraction [55]. The taxonomy of the common neural networks used in image analysis is summarized in Figure 2.

### 3.1. Inherent Characteristics and Advantages of Convolutional Neural Networks (CNNs)

Inspired by the working mechanisms of the brain, deep neural networks, also called “deep learning”, have one or more “hidden” layers between the input and output layers. In each layer, there are many neurons, also called kernels. Each kernel (usually a function in mathematics) takes inputs and computes an output. In a CNN model, a convolution kernel computes a feature at a specific location, called a “receptive field”, in the input space. The term “convolutional” denotes the operation of sliding the receptive fields through the input layer to generate the “feature map” from the convolution layer as the outputs. In essence, this operation was inspired by the functional mechanism of the visual cortex, and it makes CNN a great solution for many image analysis tasks.

A deep learning model has two important characteristics: (1) it allows for the construction and extraction of flexible representational features from input data, and (2) it contains multiple layers and many kernels that enable it to approximate basically any complex functions using the extracted features. In all, deep neural networks are capable of automatically extracting features and solving highly complex prediction problems. In contrast, traditional machine learning methods have two major steps: (1) defining the features, and (2) constructing models using these handcrafted features. Compared with traditional methods, deep learning models have the following advantages:

First, deep learning models greatly simplify or remove the task of manually defining features. Manual feature extraction is very challenging and time consuming, especially in the following two scenarios: (1) the prediction problem is complex, and/or (2) there is limited prior knowledge about the relationship between input data and the outcomes to be predicted. Both scenarios are true of pathology image analysis, as the prediction problems (such as using pathology images to predict patient outcomes or recognizing various tissue structures and cells from H&E-stained images) are very complex, and despite the accumulated knowledge from pathologists, little is known about which quantitative image features predict the outcomes. As a result, the advance of pathology image analysis had been slow and limited until the recent development of deep learning.

Second, the computation of deep learning algorithms can be highly parallel. As a result, deep learning can largely leverage the parallel computing power from the recent developments in GPU (graphics processing unit) hardware. With GPU-aided computation, processing (classifying or segmenting) a 1000 × 1000 pixels image usually takes less than one second for a deep learning model, much faster than traditional feature extraction steps [56,57] and non-deep-learning-based image segmentation methods [58]. Furthermore, since deep learning does not require handcrafted features, it can handle much more complex prediction problems and is able to recognize multiple objects simultaneously. For example, CNNs have shown great power in distinguishing as many as 1000 object categories [24].

Other advantages of deep learning methods include the following: (1) deep learning models fully utilize image data, as every pixel can be utilized in prediction model; (2) CNN models are insensitive to object position on the image, an inherent property of convolution operation; and (3) as discussed in the next section, by using extensive data augmentations in the model training process, CNN models are robust to different staining conditions in pathology image analysis.

### 3.2. Flexibility of Training and Model Construction Strategies of Deep Learning Methods

The training process of a deep learning model aims to optimize the kernels so as to minimize the difference between prediction and ground truth using the data from the training set. Traditional image processing methods usually experience difficulty in adapting to varying staining and lighting conditions [59], while deep learning models typically overcome this problem using a data augmentation strategy [24,60]. In pathology image analysis, data augmentation means the in silico creation of new training images through modifications to the color and shape of existing images. Compared with traditional image processing methods, deep-learning-based image recognition and segmentation models have shown higher stability in immunohistochemical (IHC) images [61] and H&E images, according to our experience. It is noteworthy that networks with multiple backbones provide a solution to the problem of suiting pathology images captured under different resolutions [44]. Moreover, selecting the proper image resolution depends entirely on the research goal; for example, lymphocyte and necrosis are best recognized at different scales [39].

Another advantage of deep learning methods is the flexibility of neural network construction, which consists of loss function selection and structure designation. Popular neural network frameworks, including Keras, TensorFlow, and PyTorch, have modularized the model construction process, which greatly reduces the effort required to modify and adapt an existing network. A neural network is trained by minimizing the loss function—a measure of the difference between ground truth and prediction. Although a common type of loss function for CNN is classification accuracy (e.g., differentiating tumor vs. non-malignant tissues), a wide range of loss functions can be selected to meet the needs of other problems. For example, Mobadersany et al. designed a negative partial likelihood loss function for survival outcomes in glioma and glioblastoma patients [62].

Flexibility in model architecture and structure make it relatively easy for deep learning models to integrate different sources and formats of information, such as in forming interactions between imaging and genomic features in pathology image analysis. In Mobaderany et al.’s research, the final feature layer generated from pathology images was concatenated with genomic features. This model integrated the imaging and genomic data and outperformed the model that used image data alone in predicting patient prognosis [62]. It is noteworthy that the recent developments in neural networks are making deep learning models even more powerful with increasingly complex structures. By combining a CNN with long short-term memory (LSTM) [63], another form of neural network popular in natural language processing, Zhang et al. designed a medical diagnostic model [64] that generated pathological reports and simultaneously visualized the attention of the model (i.e., which part of the input image contributed to a given word in a pathological report). This model has great potential to facilitate pathologists’ work in their daily practice.

### 3.3. Suitability for Transfer Learning

For traditional machine learning methods, applying the knowledge derived from one problem to a similar problem often requires complex mathematical deduction [65], and adapting a model developed from one dataset to a similar dataset also requires special procedures [66]. In contrast, transfer learning methods in deep learning make it straightforward and easy to adapt deep learning models developed from one dataset or problem to other similar datasets or prediction problems. Therefore, transfer learning enables us to solve some difficult prediction problems (due to the lack of a large volume of training data) by leveraging existing datasets of similar or related tasks. This is especially useful and important for biomedical applications, such as pathology image analysis, where the labeled training set is still limited. For example, the Cancer Genome Atlas (TCGA), one of the largest publicly available pathology image datasets, contains only hundreds of pathology images for each cancer type [67], while the ImageNet dataset contains 14 million labeled images for image recognition tasks. A good strategy for developing deep learning models for pathology image analysis is to start from a pre-trained model (e.g., an initialized model with pre-trained parameters from the model developed from the ImageNet dataset), and adapt the learned feature extractors to the pathology image dataset. By doing so, we can leverage the large volume of existing image datasets, such as ImageNet, for pathology image analysis. Studies have shown that this strategy leads to faster convergence and higher accuracy than training from scratch [68]. In recent years, transfer learning has been widely used in many deep learning studies of pathology image analysis tasks.

## 4. Applications of Deep Learning in Lung Cancer Pathology Image Analysis

To summarize the current progress of applying deep learning in lung cancer pathology image analysis, a survey of publications was performed. The key words “deep learning”, “pathology image”, and “lung cancer” were used in the survey. All eligible deep learning models published before October 2019 were categorized, sorted by time of publication, and are summarized in Table 1.

### 4.1. Lung Cancer Diagnosis

Several deep learning models for lung cancer diagnosis have been proposed, aiming to facilitate the work of pathologists and researchers. Since a full-size WSI is typically at the megapixel level, image patches of much smaller size (around 300 × 300 pixels) extracted from the WSI have often been used as inputs. For example, Wang et al. trained a CNN model to classify each 300 × 300 pixels image patch from H&E-stained lung ADC WSIs as malignant or non-malignant, and overall classification accuracy (malignant vs. non-malignant) was 89.8% in the testing set [69]. By applying this classification model over a sliding window across a WSI, a heatmap of the probability of being malignant for each patch could be generated to facilitate tumor detection and study tumor spatial distribution, shape, and boundary features [69]. This methodology enables fast tumor detection even when the tumor region is small, which will greatly assist pathologists in future clinical diagnoses. Similarly, Li et al. trained and compared the performance of several CNNs with different structures in classifying 256 × 256 pixels image patches as malignant vs. non-malignant [70]. In addition to malignancy detection, deep learning models have also been developed to distinguish different lung cancer subtypes. Coudray et al. trained a CNN to classify lung cancer image patches into non-malignant, ADC, or SCC [74]. Coudray et al. also trained a CNN to predict the mutation status of six frequently mutated genes in lung ADC patients based on 512 × 512 pixels pathology image patches [74]. The areas under the curve (AUCs) of the receiver operation characteristic (ROC) curves for the classification accuracy of mutated vs. non-mutated were between 0.733 and 0.856 for these six genes in the validation dataset.

### 4.2. Lung Cancer Microenvironment Analysis

As the TME is increasingly acknowledged to be an important factor affecting tumor progression and immunotherapy response, several deep learning studies have been conducted to characterize the lung cancer TME. For example, Saltz et al. developed a CNN model to distinguish lymphocytes from necrosis or other tissues at the image-patch level across multiple cancer types, including lung ADC and SCC [39]. Through quantifying the spatial organization of detected lymphocyte image patches in the WSI, Saltz et al. reported the relationships among TIL distribution patterns, prognosis, and lymphocyte fractions. Wang et al. developed a CNN model to differentiate tumor cells, stroma cells, and lymphocytes at the single-cell level in lung ADC pathology images [79]. In Wang et al.’s study, conventional image processing methods were used to extract small image patches centered on cell nuclei. The image patches were then classified into different cell types using a CNN. A prognostic model based on image features characterizing the proportion and distribution of detected cells was then developed in the training set, and prognostic performances of the model was validated in two independent datasets. Another important application for characterizing lung cancer TME is angiogenesis characterization using automatic microvessel segmentation, which has been reported to be an important prognostic factor. Yi et al. developed a microvessel segmentation model for lung ADC pathology slides using a FCN model [80]. The model also showed generalizability to breast cancer and kidney cancer slides. While manual segmentation of microvessels is laborious and prone to error, deep-learning-based segmentation is fast and can quantify the area and spatial distribution of microvessels.

### 4.3. Lung Cancer Prognosis

Predicting tumor recurrence and survival for lung cancer patients is important in treatment planning. Due to the high heterogeneity and complexity of lung cancer pathology images, predicting patient outcome from lung cancer WSI is still a very challenging problem and will require a large amount of data for model development. As a result, most studies in lung cancer prognosis that use pathology images are currently focused on developing patient outcome prediction models based on image features extracted from deep-learning-aided classification or segmentation. Wang et al. used a CNN model to segment nucleus boundaries in H&E images by classifying each pixel as nucleus centroid, nucleus boundary, or non-nucleus [83]. Nuclear morphological and textural features were then extracted and used as predictors in a recurrence prediction model, which was validated in two independent datasets, and were then reported as independent factors for early stage NSCLC recurrence after adjusting for sex and tumor stage [83]. Spatial distribution of different cell types in the TME was also reported as a prognostic factor in lung cancer. As summarized in Section 4.2, Saltz et al. [39] and Wang et al. [79] developed CNN models to classify H&E image patches as lymphocytes and other cell types in pan-cancer and lung ADC patients, respectively. The distribution patterns of different cells were reported as prognostic for both overall survival and recurrence [39,79]. In addition to nuclear- and cellular-level features, Wang et al. applied CNNs to detect tumor regions across whole-slide images and extract tumor boundary and shape features, which were reported as prognostic for lung ADC overall survival [69].

## 5. Future Directions

### 5.1. Comprehensive Lung Cancer Diagnosis and Prognosis through Multi-Task Learning

As discussed in Section 2, although lung cancer histopathological subtypes and genetic alterations have been rigorously identified and investigated, there is a lack of objective, comprehensive deep learning models able to assist pathologists in lung cancer diagnosis and prognosis. More importantly, current studies tend to develop individual models for distinct but related tasks, such as tumor detection and ADC/SCC classification. Training various models independently results in redundant effort involving shared image information across different datasets and tasks. Thus, a comprehensive deep learning model that tackles a series of lung cancer diagnosis and prognosis tasks will be of great value.

### 5.2. Interpreting Deep Learning Models and Mining Knowledge from Trained Neural Networks

Although neural networks excel in many complex tasks, they are often regarded as “black boxes”. It is challenging to interpret the model, retrieve biological information, and gain meaningful insights from a well-trained model (usually with millions of parameters). Since neural networks have proven powerful in problem solving, efforts should be made to derive biological and physiological insights from a successful model.

### 5.3. Integrating Knowledge Accumulated from Clinical and Biological Studies into Deep Learning Methods

Another important direction is to impose well-accepted knowledge into deep learning models. For example, in the case of using gene expression data to predict clinical outcomes for lung cancer patients, incorporating current knowledge of biological pathways and gene regulation networks into prediction models will likely improve model performance and interpretability. Currently, neural networks are trained based only on input data and outcome. Although the concept of “transfer learning”, which refers to borrowing knowledge from one dataset to another [65], has been raised and widely implemented, it is not straightforward to incorporate prior knowledge into deep learning models. How to incorporate knowledge into a neural network is a challenging yet popular subject in pathology image analysis, as well as in the broader AI field.

### 5.4. Utilization and Integrating Multiple Methods of Medical Imaging

Since human eyes sense color mostly in three bands (red, green, and blue), traditional cameras implemented for capturing pathology images comprise sensors for red, green, and blue lights separately. However, it is possible that other wavelength bands contain extra valuable information. Thus, imaging at multiple bands (typically 3–10 bands, known as “multispectral imaging”; imaging with narrower bands (10–20 nm) is known as “hyperspectral imaging”) has been utilized in pathological imaging analysis and reported to improve algorithm performance in image recognition [85,86]. It is anticipated that deep learning will be able to fully utilize spectral information and perform well in multispectral image recognition.

Another important direction is the integration of pathology image analysis and radiomics. Routine radiology imaging modalities, such as computed tomography (CT), positron emission tomography (PET), and magnetic resonance imaging (MRI), are crucial for cancer screening and monitoring. Rooted in radiology, radiomics focuses on extracting quantitative features by deep mining of clinical images [87,88,89]. Compared with pathology, cancer imaging in radiology is much less invasive and often captures organ- or system-level physiological and pathological features (e.g., hemodynamics based on functional MRI [90] and glucose uptake based on PET [91]). Therefore, pathological and radiological images provide complementary information for the characterization of a tumor. In addition, radiomics also benefits from the availability of high-temporal-resolution data (e.g., dynamic-contrast-enhanced CT and MRI [92]) and long-term follow-up data. AI techniques have led to promising applications in radiology, from signal processing to clinical decision assistance [93,94,95,96]. Integration of AI-powered pathology image analysis and radiomics can leverage the most significant clinical, pathological, and molecular features to ultimately improve diagnosis and prognosis [97,98]. It is noteworthy that feature extraction and dimensionality reduction are important in analyzing radiomics and genomics [99,100,101,102,103,104]. A comprehensive framework for managing and harmonizing image data and features generated from different modalities, protocols, instruments, and analysis pipelines is also anticipated to play an important role in validating and applying this integrated analytics approach [98].

## 6. Conclusions

Compared with shallow learning methods, deep learning has multiple advantages in analyzing pathology images, including simplification of feature definition, power in recognizing complex objects, time-saving through parallel computation, and suitability for transfer learning. As expected, a rapid increase in studies applying deep learning methods to lung cancer pathological image analysis has been observed. The results of relatively simple tasks, such as tumor detection and histology subtype classification, are generally satisfactory, with an AUC around 0.9, while the results of more challenging tasks, including mutation and transcription status prediction, are less satisfactory, with AUCs ranging from 0.6 to 0.8. Larger datasets, adequate neural network architecture, and modern imaging methods are anticipated to help improve the performance. Thus, combining pathological imaging with deep learning methods could have great potential impact in lung cancer patient care.

## Figures and Tables

**Table 1:** Summary of deep learning models for lung cancer pathology image analysis.

Topic	Lung Cancer Subtype	Task	Model	Prognostic Value Reported?	Year	Ref.
Lung cancer detection	ADC	Maglinant vs. non-malignant classification	CNN	Yes	2018	[69]
	NSCLC and SCLC		CNN	No	2018	[70]
	ADC and SCC		CNN	No	2019	[71]
	ADC		CNN	No	2019	[72]
	SCC		CNN	No	2019	[72]
	Not specified		CNN	No	2019	[73]
Lung cancer classification	ADC and SCC	ADC vs. SCC vs. non-malignant classification	CNN	No	2018	[74]
	ADC and SCC	Mutation status prediction	CNN	No	2018	[74]
	ADC	Histological subtype classification	CNN	No	2019	[75]
	NSCLC	PD-L1 status prediction	FCN	No	2019	[76]
	ADC and SCC	ADC vs. SCC classification	CNN	No	2019	[71]
	ADC and SCC	ADC vs. SCC classification	CNN	No	2019	[72]
	ADC and SCC	Transcriptome subtype classification	CNN	No	2019	[72]
	ADC and SCC	ADC vs. SCC vs. non-malignant classification	CNN	No	2019	[77]
	ADC	Hisotological subtype classification	CNN	No	2019	[78]
Micro-environment analysis	ADC and SCC	TIL positive vs. negative classification	CNN	Yes	2018	[39]
	ADC and SCC	Necrosis positive vs. negative classification	CNN	Yes	2018	[39]
	ADC	Tumor vs. stromal cell vs. lymphcyte classification	CNN	Yes	2018	[79]
	ADC	Microvessel segmentation	FCN	Yes	2018	[80]
	ADC	Computation staining of 6 different nuclei types	Mask-RCNN	Yes	2019	[81]
	ADC and SCC	TIL positive vs. negative classification	CNN	No	2019	[82]
Other	Early-stage NSCLC	Nucleus boundary segmentation	CNN	Yes	2017	[83]
	Not specified	Nucleus segmentation	Unet + CRF	No	2019	[84]

ADC: Adenocarcinoma; CNN: Convolutional neural network; CRF: Conditional random field; FCN: Fully convolutional neural network; Mask-RCNN: Mask-regional convolutional neural network; NSCLC: Non-small cell lung cancer; SCC: Squamous cell carcinoma; SCLC: Small cell lung cancer; TIL: Tumor-infiltrated lymphocytes.
